# Compound Fracture of the Cranium—Loss of a Portion of the Brain—Recovery

**Published:** 1846-12

**Authors:** Paul F. Eve

**Affiliations:** Professor of Surgery in the Medical College of Georgia


*Compound Fracture of the Cranium—Loss of a portion of the Brain—Recovery.* By Paul F. Eve, M.D., Professor of Surgery in the Medical College of Georgia.—In the July No., for this year, of the Provincial Medical and Surgical Journal, (England,) Mr. F. P. Smith relates a case of compound fracture of the skull in a child of four years old, who perfectly recovered, notwithstanding there had been the loss of a portion of the brain. The boy while playing under a wagon, had his head caught between the spokes of the hind wheel, and the horses starting at this moment, he was carried round and his head forced between one of the spokes and a projecting iron bolt, a space of not more than three inches and a half.

"On examining the head, I found a most frightful lacerated wound of the scalp, extending from the right temple across the forehead, and terminating in the left eye-brow; the frontal bone was laid completely bare, and the right superciliary ridge from its external to its internal angle, including a portion of the orbitar plate, was torn up. On removing this, which laid loose in the wound, I found attached to it a portion of brain, of the size of the end of the little finger, and weighing about thirty grains; four other smaller portions of bone were also removed. After cleansing the wound, I brought the flaps together, retained them so by means of several sutures, leaving a depending opening at the outer angle. Lint, dipped in cold water, was kept constantly applied over all, including the eye, which appeared to have received irremediable injury. The little patient was placed in bed, an active purgative was given, and he was confined to a milk and water diet, which was continued for a fortnight. On the following day I found he had passed a tolerably quiet night, the medicine had acted freely, and in all respects he was very much better than I expected to find him.

"To be brief, the case progressed most favorably, without a single bad symptom; in three weeks the wound had healed excepting a small opening at the outer angle; and at the end of six weeks he was playing about in his usual health. The sight of the right eye is gone and he is unable to raise the eyelid. After recovery, this boy exhibited the same restless and mischievous disposition as before the accident, which caused the loss of a portion of the cerebral substance."

Cases of recovery after portions of the brain have been lost do, occasionally occur both in civil and military practice. Some most remarkable instances will be recalled by those familiar with the records of surgery. These, however, will not vitiate the principle long established, that death is the usual result when the substance of the brain is injured in compound fractures of the cranium.

The only case where recovery took place under these circumstances within my observation, is the one about to be detailed, and the reader will remark how similar in many respects it is to the one quoted above.

A son of one of our physicians, aged six years, while leaning out of a window, fell the distance of nineteen feet upon a brick pavement. Those who saw him fall, stated he first struck his forehead, then rebounded, and was taken up for dead. This happened in September, 1840. When seen a few minutes after the accident, the left superciliary ridge or process of the os frontis was found fractured, and lying loose in the upper eyelid. It seemed to have but one attachment, and that near the nasal process of the same bone. A portion of the brain was oozing out of the lacerated wound made in the integuments near the external angle of the left eye. Our attention too, was was directed to some cerebral substance at the spot where the fall took place. I estimated the quantity of brain which escaped at the time of the accident and during the subsequent dressings to amount to a teaspoonful, or about forty grains weight. In addition to the compound fracture of the cranium with the accompanying symptoms of depression, &c., the patient sustained a dislocation of both hands backwards. These were reduced, but his father objected to the removal of the loose fragments of the superciliary process from the eyelids, or even to an attempt by pressure to replace them. The treatment pursued in the case was essentially negative, reaction spontaneously occurred without much inflammation, and in the course of a few days, the patient was sitting up.

Up to the present date, six years after the accident, Robert has continued to enjoy good health with the exception of occasional convulsions. His physical and mental faculties have been steadily developing, and he remains what he was previous to his fall, a very active restless boy. There is still a marked fullness in the left eyelid, produced by the superciliary process which is felt near its tarsal cartilage. The sight of his eye is good, but the lids often inflame and ulcerate. Above this is a large space occupied by bone, and where pulsation can be seen and felt. Pressure, or a blow, upon this spot at any time has produced convulsions, and his parents think he cannot confine his mind to close application or study.

In operating with the trephine, I have injured the brain, without unpleasant consequences to the patient.—*Southern Med. & Surg. Jour.*

